# Hymenoptera Catches of Traps with Synthetic Generic Lures from Transcarpathia (West Ukraine)

**DOI:** 10.3390/insects16090885

**Published:** 2025-08-25

**Authors:** Antal Nagy, Dóra Arnóczkyné Jakab, Attila Molnár, Zsolt Józan, Miklós Tóth, Szabolcs Szanyi

**Affiliations:** 1 Faculty of Agriculture and Food Sciences and Environmental Management, Institute of Plant Protection, University of Debrecen, 4032 Debrecen, Hungary; nagyanti@agr.unideb.hu (A.N.); jakidori6@gmail.com (D.A.J.); 2 Department of Zoology and Ecology, Hungarian University of Agriculture and Life Sciences, 2100 Gödöllő, Hungary; athoeska@gmail.com; 3 Independent Researcher, Rákóczi utca 5, 7453 Mernye, Hungary; jozan.aculeata@gmail.com; 4 Plant Protection Institute, Centre for Agricultural Research, Hungarian Research Network (HUN-REN), 1117 Budapest, Hungary; toth.miklos@atk.hun-ren.hu; 5 For the Nature- and Environmental Protection-PAPILIO (NGO), 89463 Velyka Dobron, Ukraine

**Keywords:** isoamyl alcohol, phenylacetaldehyde, lures, Apidae, Vespidae, chemical ecology

## Abstract

Between 2014 and 2016, traps baited with synthetic generic lures were used to collect data on insect diversity, with a focus on Lepidoptera pest assemblages in the surroundings of Velyka Dobron’ in Transcarpathia, West Ukraine. Traps captured a large number of insects from various taxa, including Hymenoptera, providing valuable data on both the poorly known regional fauna and the attractiveness of the lures to different Hymenoptera groups. In total, 1214 individuals were recorded, representing 11 families and 39 species, of which 17 species are reported from Transcarpathia for the first time. The majority of the sample (97%) belonged to the families Apidae and Vespidae. The Apidae and Halictidae species were predominantly attracted to phenylacetaldehyde-based lures, while Vespidae species responded more to semisynthetic lures containing isoamyl alcohol. The parallel use of the tested lures with different selectivity appears to be an effective, user-friendly, standardized method for studying Hymenoptera assemblages. Furthermore, knowledge of lure selectivity allows for the target collection of species belonging to different ecotypes.

## Simple Summary

Samplings carried out in Velika Dobron’, West Ukraine, between 2014 and 2016 using volatile-baited traps provided data on insect biodiversity, including Hymenoptera. Of the 39 Hymenoptera species recorded, 17 are reported for Transcarpathia for the first time, representing a significant contribution to the knowledge of the regional fauna. Furthermore, the tested phenylacetaldehyde- and isoamyl alcohol-based lures showed attractiveness and different selectivity to different Hymenoptera taxa. These findings support the parallel use of such lures in standardized quantitative surveys and in targeted sampling of specific Hymenoptera taxa.

## 1. Introduction

In field studies, different sampling methods can be used to collect Hymenoptera species depending on the goals and targeted taxa. The most widely used methods are the pan and Malaise traps and the more efficient but more labor-intensive transect count with an insect net. With combined use and modification of these methods (e.g., use of different pan-trap colors and net types), the efficiency can be improved considering both abundance and number of species caught [1,2,3,4,5,6]. Additionally, many valuable data may be collected in studies targeting other taxa; thus, collecting and reviewing them may also be a useful tool for hymenopterology. These data may originate from samplings carried out with traps including different generic lures, attracting a wide range of insect taxa [7,8,9,10,11]. Lures contain different semiochemicals that attract several taxa, including economically important pests and even protected species. These compounds attract species with the same food source preferences and may be useful and effective in monitoring and control. Their use in biodiversity assessment and ecological entomology, and in assessing their non-target catches, is less common [12,13].

In the present study, a synthetic phenylacetaldehyde-based lure containing compounds of flower scents (FLOs) and a semi-synthetic isoamyl alcohol-based lure (SBL) referring to fermented liquids were used primarily to monitor Lepidoptera pest species and additionally to assess insect biodiversity of the studied region. Results on the Lepidoptera sampling were published in 2017 and 2024 [14,15], while those on other taxa were previously evaluated (Diptera [16], Neuroptera [17], Orthoptera [18]). The attractivity of phenylacetaldehyde to *Apis mellifera* L., *Bombus terrestris* (L.), and some species of Halictidae is already known [19]. Isoamyl alcohol is a component of the alarm pheromone of *Vespa mandarinia* (S.). This pheromone consists of three volatiles (2-pentanol, isoamyl alcohol, and 2-pentyl-isovalerate), but none of them has a significant effect alone; thus, the isoamyl alcohol acts as a synergistic compound in this mixture [20,21]. Further studies on the effect of the mentioned compounds on hymenopterans may help to understand the mode of their action and can help with the harmonization of the goals of plant protection and environmental protection.

The Hymenoptera fauna of the Bereg Lowland is poorly known [22,23,24,25,26]. Although there were some studies on the neighboring Ukrainian Carpathians and the connected lowland regions [27,28,29,30,31,32,33,34,35,36,37,38,39,40,41,42,43,44,45], for the last decade data have not been published. Since the area now is nearly unavailable to foreign researchers, the data provided by non-target catches of baited traps are gap-filling and are nearly the only recent data from the area.

## 2. Materials and Methods

### 2.1. Sampling Area

The samplings were conducted in the margin of the Game Reserve of Velyka Dobron’, which is located on the edge of the former Szernye peatland near the village in West Ukraine (GPS: 48.4338° N, 22.4041° E). The native wildlife of the former peatland was extremely diverse [46], but the most valuable relict habitats have become extinct. Recently, the area has been dominated by secondary habitats with fragments of the original wetlands and forests. The remains of the oak–ash–elm hardwood gallery forest represents the most native and valuable habitat type of the reserve. The canopy is formed mainly by *Quercus robur*, *Fraxinus angustifolia* subsp. *pannonica*, *Ulmus laevis*, *Populus canescens*, and *Frangula alnus*. The also-valuable pedunculate oak–hornbeam forest is rich in geophytic species (e.g., *Scilla drunensis*, *Anemone nemorosa*, *A. ranunculoides*, etc.) and dominated by *Q. robur* and *Carpinus betulus*. Other natural and semi-natural habitats are the rather xeric silver lime—oak forests and forest fringes, tall forb forest fringes, and willow scrubs. The forest clearings maintain the remains of natural sites of forest edges and meadows. The reserve is surrounded by agricultural lands protected by drainage channels.

### 2.2. Trapping

The detailed composition of the lures was published, e.g., in Szanyi et al. [15] and Nagy et al. [16,18].

CSALOMON^®^ VARL+ funnel traps (HUN-REN ARC Plant Protection Institute, Budapest, Hungary)—photos of the trap can be viewed at www.csalomontraps.com (accessed on 28 February 2025)—were used baited with synthetic compound previously isolated and identified from fermenting liquids (=SBL) [15] or with synthetic floral compounds (=FLOs), which had previously been isolated and identified from the scent of several flowers [15,21,47].

The SBL lure was a mixture of isoamyl alcohol, acetic acid, and red wine (1:1:1; 3 mL) that was administered on a dental roll inside a polypropylene tube with 4 mL capacity [15]. The compounds could evaporate across a smaller opening with a 4 mm diameter, which was opened in the field. The wine was a cuvee (cellar of Dr. G. VÖRÖS) including Bluefrankish (70%), Merlot (15%), Kadarka (7.5%), and Blauburger (7.5%), with 13.6–13.8% alcohol and 0.4-0.6 g/L volatile acid (acetic acid). The FLO lure contained phenylacetaldehyde, (*E*)-anethol, benzyl acetate, and eugenol (1:1:1:1) [15,47]. The lure was placed inside polyethylene bag dispensers [47]. Synthetic compounds (>95% purity) applied in baits were obtained from Sigma-Aldrich Kft. (Budapest, Hungary).

To kill the captured insects, a small piece (1 × 1 cm) of a household anti-moth insecticide strip (Chemotox^®^ SaraLee, Temana Intl. Ltd., Slouth, UK) containing 15% dichlorvos as active ingredient, was placed inside the trap container.

Traps were placed in a diverse habitat consisting of extensive agricultural lands, remains of the former peatland and oak–hornbeam gallery forests [46]. Both trap types were used in four repetitions, i.e., 4 × 2 traps were placed on the trees of the sampling site 1.8–2 m high and 20 m from each other. The traps were deployed between 20 June and 19 October in 2014, between 24 May and 11 October in 2015, and between 10 April and 11 September in 2016. They were checked and emptied once a week and were rotated to eliminate the local effects. The samples were stored deep-frozen, then they were separated by taxa and were identified.

### 2.3. Evaluation of Data

To evaluate the catches, a checklist of the species was compiled, the numbers of individuals caught were counted by family and species, and the relative frequencies were also calculated separately for both tested types of lures and for the whole sample. The attractiveness of the tested lures was assessed based on the number and ratio of species caught, as well as the relative frequencies of taxa. The relative frequency (RF) of each species was quantified as the proportion of sampling units in which the species was present, expressed as a percentage. Specifically, RF was computed using the following formula: RF = (number of sampling units in which the species occurred/total number of sampling units) × 100. For data processing, visualisation and statistical analysis MS Office 365 program packages were used.

The selectivity of the lures was characterized by the qualitative and quantitative composition of the summarized samples caught by them.

## 3. Results

During the three-year study, 1214 individuals (N) belonging to 39 species (S) of 11 families of Hymenoptera were caught. The most species-rich families were Apidae (10 species), Vespidae (9 species), Andrenidae, and Halictidae (6 species), while Colletidae, Mellitidae, and Pompilidae were represented with only a single species per family. Seventeen species, which was 43.6% of the species caught, had not been previously reported from Transcarpathia [48,49,50,51,52]. The six Haltictidae and the two Crabronidae species belonged to this group (Table 1 and Table 2).

Beyond the honeybees, 38 wild Hymenoptera species were caught, which was 97.4% of all the fauna sampled. The most abundant species was the honeybee, with 31.9% relative frequency and continuous presence. Among wild hymenopterans, other Apidae, with 219 individuals (RF = 18.0%), and Vespidae (RF = 45.6%) were the most abundant groups. The five most dominant species were *Apis mellifera* (31.9%), *Vespula germanica* (23.6%), *Vespa crabro* (18.0%), *Bombus terrestris* (11.0%), and *B. hortorum* (4.3%) (Table 2).

The total relative frequency of wild Hymenopterans was 68.1%, from which flower-visitors (26 species) comprised 21.7% total relative frequency of the whole sample.

### Response of the Hymenoptera Taxa to the Tested Lures

The two tested lures showed different attractivity to the Hymenoptera taxa caught. Considering the number of species caught, the efficiency of the FLO lure was higher since it caught more than three times as many species as the SBL lure (34 to 11). However, the FLO lure attracted slightly more individuals, so from this point of view, the efficiency of the two tested lure can be seen as similar (Table 2).

**Figure 1:**
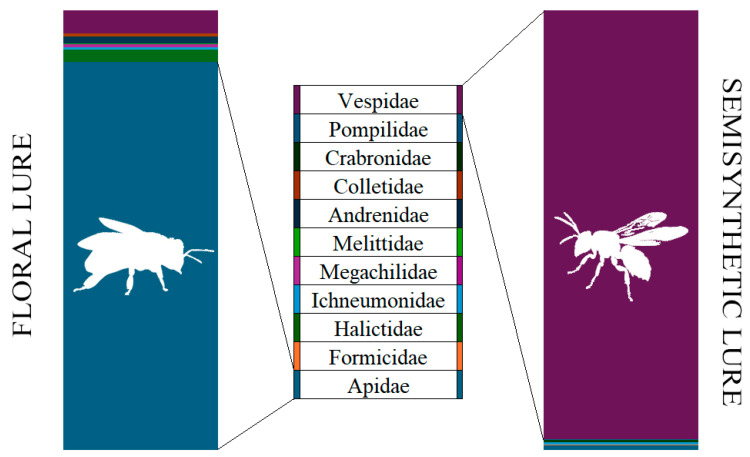
Relative frequencies (%) of Hymenoptera families caught by the two tested lures.

Species belonging to Andrenidae, Apidae, Colletidae, Halictidae, Megachilidae, and Melittidae were exclusively or predominantly attracted to the phenylacetaldehyde-based FLO lure, with a ratio of 99.0–100.0%. In contrast, all individuals of Crabronidae and Pompilidae (100.0%), as well as a high ratio of Vespidae specimens (93.7%), were found in the SBL traps. The volatile preferences of the Apidae and Vespidae families were the most conspicuous considering the relative frequencies of Hymenoptera species attracted to different lures (Figure 1).

In the Vespidae family, the less abundant species of Eumeninae (*D. zonalis* and *S. gracilis*) and *Polistes dominula* were caught only with the FLO traps. This resulted in a higher number of Vespidae species in the FLO traps than in the SBL traps; however, the total abundances were significantly higher in the SBL traps because of the preferences of highly abundant *Vespa* spp. (Table 1).

## 4. Discussion

Our results show that the native wildlife of the region has high diversity, as was proven also in the case of lepidopterans [14,15], coleopterans [53], trichopterans [54,55], orthopterans [56,57], and the flora [58].

Our study provides a foundation for compiling a complete checklist of the Hymenoptera fauna of Transcarpathia, which has not been edited so far, and only sporadic data have been published from the region [27,28,30,31,36,37,38,41,43,44]. Although the Hungarian part of the region has been more extensively studied [23,24,25], our data contribute valuable information toward understanding the Hymenoptera fauna of the Hungarian–Ukrainian cross-border region of the Bereg Lowland.

Our data address the existing gap in knowledge regarding volatile compounds attractive to Hymenopterans, as the efficacy of only a few semiochemicals has been proven to date [21] and comparative studies among them have not yet been conducted. The revealed differences in the lures’ attractivity referred to the different food preferences of the taxa caught. The FLO lure containing compounds of flower scent attracted flower-visiting taxa (e.g., Apidae, Halictidae), while the SBL lure, resembling fermented liquids, mainly attracted species belonging to the Vespidae family (Table 1 and Table 2). Although the efficiency of the tested lures may be lower than that of some other methods generally used in hymenopterology (e.g., Malaise traps), the high number of newly reported species showed the advantages of the method for species diversity assessment, and it is worth using them parallelly as supplementary methods.

The revealed specificity of the lures studied provides opportunities for selective samplings of Hymenoptera taxa with different food source preferences. Additionally, through modification of the funnel traps using nets in the upper opening of the funnel, the selectivity may be increased regarding the size of the targeted taxa, since more dense nets can exclude larger species from the traps. On the other hand, when using these lures in other types of insect traps, their effect range and efficiency may be modified and/or increased.

These results may serve as a basis for further investigations of volatile preferences of different Hymenoptera taxa and may help to develop a new, easy-to-use, standardizable trapping method for faunistical and ecological studies.

## Figures and Tables

**Table 1:** List of the Hymenoptera species caught between 2014 and 2016 in Velyka Dobron’, West Ukraine, by volatile traps, with their abundances and temporal constancy (number of individuals caught/number of years when the species was caught), and their relative frequency (RF%) in the whole sample. * = Species formerly not reported from Transcarpathia.

Species	Family	SBL	FLO	N. of Ind.	RF%
*Andrena flavipes* (Panzer, 1799)	Andrenidae		2/1	2	0.16
*Andrena fulvata* (Stöckhert, 1930) *	Andrenidae		3/1	3	0.25
*Andrena gravida* (Imhoff, 1832)	Andrenidae		1/1	1	0.08
*Andrena nitida* (Müller, 1776)	Andrenidae		2/1	2	0.16
*Andrena praecox* (Scopoli, 1763)	Andrenidae		1/1	1	0.08
*Andrena rufula* (Schmiedeknecht, 1883)	Andrenidae		2/2	2	0.16
*Apis mellifera* (Linnaeus, 1758)	Apidae	4/3	383/3	387	31.88
*Bombus haematurus* (Kriechbaumer, 1870)	Apidae		6/2	6	0.49
*Bombus hortorum* (Linnaeus, 1761)	Apidae	2/1	50/3	52	4.28
*Bombus hypnorum* (Linnaeus, 1758)	Apidae		1/1	1	0.08
*Bombus muscorum* (Linnaeus, 1758) *	Apidae		1/1	1	0.08
*Bombus pascuorum* (Scopoli, 1763)	Apidae		23/3	23	1.89
*Bombus sylvarum* (Linnaeus,1761)	Apidae		3/1	3	0.25
*Bombus terrestris* (Linnaeus, 1758)	Apidae		133/3	133	10.96
*Nomada goodeniana* (Kirby, 1802)	Apidae		1/1	1	0.08
*Nomada panzeri* (Lepeletier, 1841)	Apidae		1/1	1	0.08
*Colletes cunicularius* (Linnaeus,1761) *	Colletidae		5/1	5	0.41
*Mimumesa beaumonti* (van Lith, 1949) *	Crabronidae	2/1		2	0.16
*Trypoxylon figulus* (Linnaeus,1758) *	Crabronidae	1/1		1	0.08
*Lasioglossum calceatum* (Scopoli, 1763) *	Halictidae		3/2	3	0.25
*Lasioglossum lativentre* (Schenck, 1853) *	Halictidae		1/1	1	0.08
*Lasioglossum marginatum* (Brullé, 1832) *	Halictidae		11/2	11	0.91
*Lasioglossum morio* (Fabricius, 1793) *	Halictidae		3/2	3	0.25
*Lasioglossum pauxillum* (Schenck, 1853) *	Halictidae		1/1	1	0.08
*Lasioglossum punctatissimum* (Schenck, 1853) *	Halictidae		1/1	1	0.08
*Megachile centuncularis* (Linnaeus, 1758) *	Megachilidae		1/1	1	0.08
*Osmia bicolor* (Schrank,1781)	Megachilidae		1/1	1	0.08
*Osmia cornuta* (Latreille, 1805)	Megachilidae		3/1	3	0.25
*Macropis europaea* (Warncke, 1973) *	Melittidae		1/1	1	0.08
*Priocnemis melanosoma* (Kohl, 1880) *	Pompilidae	1/1		1	0.08
*Ancistrocerus nigricornis* (Curtis, 1826) *	Vespidae	1/1		1	0.08
*Discoelius zonalis* (Panzer, 1801) *	Vespidae		1/1	1	0.08
*Dolichovespula media* (Retzius, 1783) *	Vespidae	15/3	5/2	20	1.65
*Polistes dominulus* (Christ, 1791)	Vespidae		1/1	1	0.08
*Polistes nimphus* (Christ, 1791)	Vespidae	2/1	1/1	3	0.25
*Symmorphus gracilis* (Brullé, 1832)	Vespidae		1/1	1	0.08
*Vespa crabro* (Linnaeus, 1758)	Vespidae	206/3	13/1	219	18.04
*Vespula germanica* (Fabricius, 1793)	Vespidae	273/3	13/2	286	23.56
*Vespula vulgaris* (Linnaeus, 1758)	Vespidae	21/2		21	1.73
	Formicidae	1/1		1	0.08
	Ichneumonidae	3/1	3/1+	6	0.49
Sum				1214	

**Table 2:** Number of species caught (S) belonging to different families and their abundance (N) by lures (SBL and FLO) and in total, their relative frequency (%) in the whole sample, and the ratio (%) of the individuals caught in traps baited with different lures.

Family	S (Total)	N (Total)	FR%	S		N		N (Ratio%)	
				FLO	SBL	FLO	SBL	FLO	SBL
Andrenidae	6	11	0.9	6	0	11	0	100.0	0.0
Apidae	10	608	50.1	10	2	602	6	99.0	1.0
Colletidae	1	5	0.4	1	0	5	0	100.0	0.0
Crabronidae	2	3	0.2	0	2	0	3	0.0	100.0
Halictidae	6	20	1.6	6	0	20	0	100.0	0.0
Megachilidae	3	5	0.4	3	0	5	0	100.0	0.0
Melittidae	1	1	0.1	1	0	1	0	100.0	0.0
Pompilidae	1	1	0.1	0	1	0	1	0.0	100.0
Vespidae	9	553	45.6	7	6	35	518	6.3	93.7
**Sum (sp.)**	**39**	**1207**	**99.4**	**34**	**11**	**682**	**532**	**56.5**	**43.5**
Formicidae	-	1	0.1	-	-	0	1	0.0	100.0
Ichneumonidae	-	6	0.5	-	-	3	3	50.0	50.0
**Sum (total)**		**1214**		**-**	**-**	**685**	**536**	**56.4**	**43.6**

## Data Availability

The raw data supporting the conclusions of this article will be made available by the authors on request.
